# Evaluation of Serum Ferritin, Procalcitonin, and C-Reactive Protein for the Prediction of Severity and Mortality in Hemorrhagic Fever With Renal Syndrome

**DOI:** 10.3389/fmicb.2022.865233

**Published:** 2022-05-23

**Authors:** Lihe Che, Zedong Wang, Na Du, Liang Li, Yinghua Zhao, Kaiyu Zhang, Quan Liu

**Affiliations:** ^1^Changchun Veterinary Research Institute, Chinese Academy of Agricultural Sciences, Changchun, China; ^2^Key Laboratory of Organ Regeneration and Transplantation of the Ministry of Education, Center for Pathogen Biology and Infectious Diseases, The First Hospital of Jilin University, Changchun, China; ^3^Department of Infectious Diseases, The First Hospital of Jilin University, Changchun, China; ^4^School of Life Sciences and Engineering, Foshan University, Foshan, China

**Keywords:** hantaviruses, serum ferritin, hemorrhagic fever with renal syndrome, C-reactive protein, procalcitonin

## Abstract

This study aimed to analyze the clinical significance of serum ferritin, procalcitonin (PCT), and C-reactive protein (CRP) in patients with hemorrhagic fever with renal syndrome (HFRS). The demographical, clinical, and laboratory data of 373 patients with HFRS in northeastern China were retrospectively analyzed. The levels of serum ferritin and PCT in severe patients (*n* = 108) were significantly higher than those in mild patients (*n* = 265, *p* < 0.001) and associated with HFRS severity. The area under the receiver operating characteristic curve (AUC) values of serum ferritin and PCT for predicting the severity of HFRS were 0.732 (95% CI 0.678–0.786, *p* < 0.001) and 0.824 (95% CI 0.773–0.875, *p* < 0.001), respectively, showing sensitivity and specificity of 0.75 and 0.88 for serum ferritin, and 0.76 and 0.60 for PCT. The CRP level in HFRS with bacterial co-infection (*n* = 115) was higher than that without bacterial co-infection (*n* = 258, *p* < 0.001). The AUC value of CRP for predicting bacterial co-infection was 0.588 (95% CI 0.525–0.652, *p* < 0.001), showing sensitivity and specificity of 0.43 and 0.76, respectively. The serum ferritin level in non-survivors (*n* = 14) was significantly higher than in survivors (*n* = 359, *p* < 0.001). The AUC value of serum ferritin for predicting mortality was 0.853 (95% CI 0.774–0.933, *p* < 0.001), showing sensitivity and specificity of 0.933 and 0.739. Serum ferritin and PCT have a robust association with HFRS severity and mortality, which may be promising predictors, and CRP is an effective biomarker to assess bacterial co-infection in HFRS.

## Introduction

Hemorrhagic fever with renal syndrome (HFRS) is caused by Hantaviruses of the genus *Orthohantavirus* in the family *Hantaviridae* and is characterized by acute kidney injury, increased vascular permeability, and coagulation abnormalities. HFRS is an important rodent-associated zoonosis endemic in eastern Asia, especially in China, accounting for ∼90% of the cases globally (Jonsson et al., 2010). Hantaan virus and Seoul virus are the major etiologies of HFRS in China that cause severe and mild forms of HFRS, respectively (Zhang et al., 2010). Although effective treatment and vaccination have greatly reduced the impact of HFRS in the past few decades in China, the number of infected patients still remains at a relatively high level, with ∼10,000 cases reported annually. Clinical parameters are necessary for improving the management of patients with HFRS, which may be used as clinical references for severity assessment and prognosis prediction (Du et al., 2014; Zhang et al., 2015).

Cytokine storm of excessive immune response plays a central role in the pathogenesis of HFRS, which can damage the vascular endothelial cell and accelerate platelet activation, leading to multi-organ dysfunction or failure (Wang et al., 2012). The pathophysiology of organ damage that occurs with hantavirus infection also motivates the increase in biomarkers, including ferritin, CRP, and PCT. Ferritin has been identified as an important molecule in the host immune system that can reflect the cellular defense against inflammatory response (Valero et al., 2019). Studies also have confirmed ferritin as a potential biomarker in the diagnosis of viral and bacterial infection, as well as macrophage-activation syndrome and hemophagocytic lymphohistiocytosis (Barut et al., 2010; Kyriazopoulou et al., 2017; Franco-Martinez et al., 2021; Zhou J. et al., 2021). Patients with HFRS appear to have a higher level of serum ferritin and PCT (Tjendra et al., 2020). Procalcitonin (PCT) is a precursor of calcitonin hormone secreted by different cells under inflammatory stimulation, which is increased and associated with disease severity, secondary bacterial infection, and mortality in patients with HFRS (Fan X. et al., 2018). C-reactive protein (CRP) is a sensitive biomarker of viral infection and inflammation (Shin et al., 2018). CRP and PCT can be used to evaluate disease severity and predict outcomes in patients with COVID-19 (Liu et al., 2020; Pan et al., 2020; Zhang et al., 2020, 2021; Zhou Y.Z. et al., 2021). In this study, we aimed to analyze the clinical significance of serum ferritin, procalcitonin (PCT), and C-reactive protein (CRP) in patients with HFRS.

## Materials and Methods

### Study Design

We collected the demographical, laboratory, and clinical data of patients with HFRS who were admitted to The First Hospital of Jilin University during 2018–2019. Patients who were hantavirus (HV) serological IgM positive, had typical clinical manifestations of febrile, hemorrhage, renal injury, and travel history to HV epidemiology areas were diagnosed with HFRS and included in this study. Exclusion criteria included other infections similar to HFRS, hantaviruses IgM negative, or acute kidney injury due to other diseases (e.g., dehydration, hemorrhagic shock, glomerulonephritis, and acute intoxication).

To investigate the change of serum ferritin, PCT, and CRP associated with severity, bacterial co-infection, and death due to HFRS, we divided the patients into two groups according to the classification criteria (Escutenaire and Pastoret, 2000; Liu et al., 2008; Brocato and Hooper, 2019). The clinical types were categorized into four groups (mild, moderate, severe, and critical) according to the severity of hantavirus infection (Table 1). To understand the relationship between serum ferritin, PCT, CRP, and hantavirus infection more clearly, we divided the patients into two groups (mild group: mild and moderate type patients, and severe group: severe and critical type patients).

**TABLE 1:** Standard for the classification of HFRS severity.

Clinical types	Manifestations*
Mild	*T* < 39°C; mild toxemic symptoms and renal damage; no bleeding except for hemorrhagic spots, no shock and oliguria
Medium	*T* = 39°C∼40°C; severe toxemic symptoms and obvious chemosis; The systolic Bp < 90 mmHg, or pulse pressure < 26 mmHg during the whole clinical course; Obvious hemorrhage and oliguria, urinary protein “+++”
Severe	*T* > 40°C; severe toxemic symptoms and effusion, some may have toxemic psychiatric symptoms; Shock; Severe ecchymoses on skin and cavity hemorrhage; Oliguria lasting for <5 days or anuresis less than 2 days
Critical	Combining one of the following on severe style: Refractory shock; Hemorrhage of important organs; Oliguria lasting for >5 days, or anuresis for >2 days or urea nitrogen >120 mg/dl (42.84 mmol/L); Heart failure or pulmonary edema; Central nervous system damage such as cerebral edema, brain hemorrhage and cerebral hernia; Severe secondary infections

*T, body temperature; BP, blood pressure.

### Clinical Diagnosis and Data Collection

The clinical information of HFRS included the patients’ age, sex, admission days after fever, days in the hospital, primary disease or comorbidity, complication caused by HFRS, such as hemorrhage, bacterial co-infection, acute respiratory distress syndrome (ARDS), and multiple-organ dysfunction syndrome (MODS). Clinical symptoms, including hemoptysis, hematemesis, melena, hematuria, ecchymosis, and rupture or bleeding of internal organs of the body, were diagnosed with hemorrhage. The bacterial co-infection, including *Staphylococcus aureus*, methicillin-resistant *S. aureus, Streptococcus pneumoniae, Klebsiella pneumoniae, Pseudomonas aeruginosa*, and *Enterobacter aerogenes*, was confirmed by liquid culture assay (blood, urine, or feces) combined with higher levels of CRP, PCT, and/or radiological evidence. ARDS and MODS were diagnosed according to the criteria (Ramirez, 2013; Fan E. et al., 2018).

Serum ferritin, PCT, and CRP were detected at least three times at the same timepoint (hospital admission, the third day in hospital, and the day patients were discharged or bacterial co-infection was detected) from the first day of admission to the last day. Other laboratory results, such as white blood cell (WBC), platelet (PLT), alanine transaminase (ALT), aspartate aminotransferase (AST), lactate dehydrogenase (LDH), and α-hydroxybutyrate dehydrogenase (α-HBDH), were detected at least once at a different stage of HFRS. All the patients with HFRS received ribavirin and supportive treatment (maintaining fluid and electrolyte balance).

### Statistical Analysis

Statistical analysis was performed using SAS 9.3 software. Categorical data were presented as numbers and percentages, and analyzed using Pearson’s Chi-square test or Fisher’s Exact. Distributed data were described using mean and standard deviation and analyzed using Student’s *t*-test. Non-parametric data were analyzed using the Mann–Whitney *U* test, shown as median and interquartile range (IQR). Results with a value of *p* < 0.05 were deemed as statistically significant. To make data conform to normality and reduce the variability, we used log transformation, especially in data sets that included outlying observations. Predictive values of variables for disease severity, bacterial co-infection, or prognosis were tested with receiver operating characteristic (ROC) curves and quantified by calculating the area under the ROC curve (AUC) and the 95% confidence interval (CI) using SPSS 19.0 software (SPSS Inc., Chicago, IL, United States; Fan X. et al., 2018).

## Results

### Clinical Information

The study included 373 patients with HFRS (286 men and 87 women) with a mean age of 45.38 ± 14.13 years; 108 patients were grouped into severe types (97 critical forms and 11 severe forms) and 265 patients were grouped into mild types (47 mild forms and 218 medium forms). Non-bacterial co-infections included 258 patients, while bacterial co-infections included 115 patients. Survivors included 359 patients and 14 died of HFRS. Patients who died of HFRS were all in the severe group (*p* < 0.001). The patients in the severe group had higher frequencies of complication of hemorrhage (*p* < 0.001), hepatic injury (ALT > 50 U/L and AST > 40 U/L) (*p* < 0.001), ARDS (*p* < 0.001), MODS (*p* < 0.001) than those of the mild group. Continuous renal replacement therapy (CRRT) and respirators were used in the severe group (*p* < 0.001). The patients in the severe group had a longer hospital stay than those in the mild group (*p* < 0.001), but there was no significant difference in incubation between the two groups. Higher WBC and lower PLT in the severe group were other characteristics (*p* = 0.147 and *p* < 0.001, respectively). The patients in the severe group had more severe hepatic and myocardial injury than those in the mild group.

### Serum Ferritin, Procalcitonin, and C-Reactive Protein Association With Severity of Virus Infection

In the 373 patients with HFRS, the median serum ferritin level was 3620.3 ng/ml, ranging from 70.1 to 117,500 ng/ml. The median serum ferritin level in the severe group was higher than that of the mild group (*p* < 0.001, Table 2). The median PCT and CRP levels in these patients with HFRS were 1.84 (0.02–82.92) ng/ml and 32 (1.25–212) mg/l, respectively, which were higher than the reference value of less than 0.5 ng/ml and 10 mg/l; both PCT and CRP in the severe group were higher than those of the mild group (*p* < 0.001; Table 2).

**TABLE 2:** Demographics and clinical and laboratory data at admission in the patients with HFRS of different clinical types*.

Variables	Mild (*n* = 265)	Severe (*n* = 108)	*P*-value
Male, n (%)	208 (78.5)	78 (72.2)	0.1975
Age, years	45 (32–54)	49.3 ± 12.6	0.0009
Time from symptom onset to hospital admission, days	5 (4–7)	5 (4–6)	0.0353
Hospital stay, days	7 (5–9)	9 (7–13)	<0.0001
**Comorbidity**			
Hypertension, n (%)	10 (3.8)	11 (10.2)	0.0148
Coronary heart disease, n (%)	2 (0.8)	2 (1.9)	0.7048
Diabetes, n (%)	8 (3.0)	10 (9.3)	0.0108
**Complication**			
Hemorrhage, n (%)	34 (12.8)	71 (65.7)	<0.0001
Hepatic injury, n (%)	169 (63.8)	90 (83.3)	0.0002
Myocardial damage, n (%)	80 (30.2)	76 (70.4)	<0.0001
Bacterial infection, n (%)	78 (29.4)	37 (34.3)	0.3600
ARDS, n (%)	0	19 (17.6)	<0.0001
MODS, n (%)	0	21 (19.4)	<0.0001
Respiratory support, n (%)	0	35 (32.4)	<0.0001
CRRT, n (%)	0	42 (38.9)	<0.0001
Number of death, n (%)	0	14 (13.0)	<0.0001
**Parameters**			
WBC, × 10^9^/L	8.4 (5.3–10.8)	9.6 (5.5–11.5)	0.1407
PLT, × 10^9^/L	109.0 (88.0–125.0)	39.0 (20.0–85.0)	<0.0001
AST, U/L	88.8 (56.0–155.6)	149.4 (77.5–297.4)	<0.0001
ALT, U/L	109.1 (68.9–187.0)	88.0 (67.9–173.2)	0.2602
LDH, U/L	415.0 (344.0–536.0)	655.0 (415.0–1011.0)	<0.0001
α-HBDH, U/L	303.0 (247.0–376.5)	515.0 (340.0–804.0)	<0.0001
PCT, ng/ml	1.3 (0.8–2.1)	3.8 (2.4–6.8)	<0.0001
Ferritin, ng/ml	2502.4 (1237.7–6670.5)	9930.5 (3604.3–17080.0)	<0.0001
CRP, mg/l	29.5 (16.4–53.2)	42.1 (25.8–69.4)	0.0006
Lg PCT	0.1 (−0.1 to 0.3)	0.6 (0.4–0.8)	<0.0001
Lg Ferritin	3.4 (3.1–3.8)	4.0 (3.6–4.2)	<0.0001
Lg CRP	1.5 ± 0.4	1.6 (1.4–1.8)	0.0006

*ARDS, acute respiratory distress syndrome; MODS, multiple organ dysfunction syndrome; CRRT, continuous renal replacement therapy; WBC, white blood cell count; PLT, platelet; ALT, alanine transaminase; AST, aspartate aminotransferase; LDH, lactate dehydrogenase; and α-HBDH, α-hydroxybutyrate dehydrogenase; Lg Ferritin, Lg PCT, and Lg CRP, Ferritin, procalcitonin, and CRP after log10 transformation.

In 258 patients without bacterial co-infection (73 patients in the severe group and 185 patients in the mild group), the median serum ferritin level of the severe group [976 ng/ml (range 219–3,683.3 ng/ml)] was higher than that of mild [778.4 ng/ml (range 70.1–5,310 ng/ml)]. The median PCT level of the severe group [1.26 ng/ml (range 0.02–12.93 ng/ml)] was higher than that of the mild group [0.99 ng/ml (range 0.08–41.3 ng/ml)]. The median CRP level of the severe group [32.5 mg/l (range 3.11–509.9 mg/l)] was higher than that of the mild group [24.9 mg/l (range 1.25–167 mg/l)], although the difference was not statistically significant.

In the 21 patients with MODS (all in the severe group), the median levels of serum ferritin, PCT, and CRP were 33,070 ng/ml (range 20,691–99,800 ng/ml), 4.13 ng/ml (range 0.26–56 ng/ml), and 49.4 mg/l (range 8.78–127 mg/l), respectively; in the 19 patients with ARDS (all in the severe group), the median levels of serum ferritin, PCT, and CRP were 18,511 ng/ml (range 10,550–99,800 ng/ml), 5.0 ng/ml (range 0.02–82.92 ng/ml), and 55.89 mg/l (range 5.02–145 mg/l), respectively.

**FIGURE 1:**
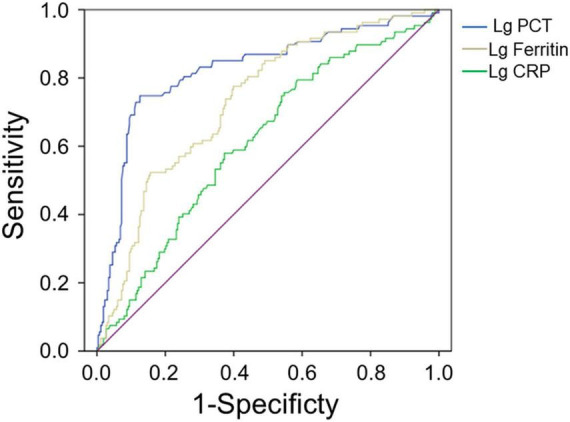
Performance of serum ferritin, PCT, and CRP as predictors of severity by receiver operating characteristics curve analysis. The AUC of serum ferritin = 0.732, the AUC of PCT = 0.824, and the AUC of CRP = 0.614, all *p* < 0.01.

The AUC value of serum ferritin for predicting the severity of HFRS was 0.732 (95% CI 0.678–0.786, *p* < 0.001), while PCT and CRP for predicting the severity of HFRS were 0.824 (95% CI 0.773–0.875, *p* < 0.001) and 0.614 (95% CI 0.552–0.676, *p* = 0.001), respectively (Figure 1 and Table 3), showing that PCT and ferritin may be potential biomarkers to predict the severity of HFRS.

**TABLE 3:** Predictive values of parameters for the severity of HFRS*.

Variables	AUC	*p* value	Cut-off value	Sensitivity	Specificity	95% CI for AUC	
						
						Lower	Upper
Lg PCT	0.824	<0.001	0.45	0.75	0.88	0.773	0.875
Lg CRP	0.614	0.001	1.41	0.76	0.45	0.552	0.676
Lg Ferritin	0.732	<0.001	3.55	0.76	0.60	0.678	0.786

*AUC, area under the receiver operating characteristic curve; CI, confidence interval; Lg PCT, procalcitonin after log10 transformation; Lg CRP, C-reactive protein after log10 transformation; Lg Ferritin, serum ferritin after log10 transformation.

### Serum Ferritin, Procalcitonin, and C-Reactive Protein Association With Bacterial Co-infection

In 373 patients with HFRS, 115 patients combined bacterial co-infection (37 patients in severe group and 78 patients in mild group). The incidence of secondary bacterial co-infection of patients in the hospital was 30.8%, generally occurring on days 7–10 of the virus infection. The level of serum ferritin in patients with bacterial co-infection was significantly higher than that in patients with non-bacterial co-infection. The median serum ferritin level in 115 patients with bacterial co-infection [2,799.0 ng/ml (range 1,542.8–5,941.7 ng/ml)] was higher than that in 258 patients with non-bacterial co-infection [807.85 ng/ml (range 515.1–1,358.8 ng/ml)] (*p* < 0.001, Table 3). The AUC value of serum ferritin for predicting bacterial co-infection was 0.54 (95% CI 0.477–0.602, *p* = 0.214).

**FIGURE 2:**
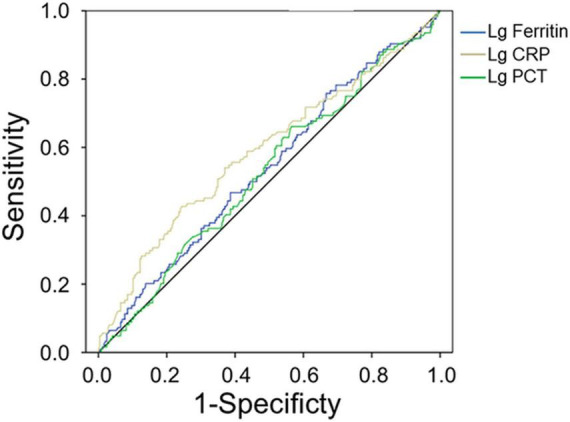
Receiver operating characteristics curve showing the performance of serum ferritin as a predictor of bacterial co-infection. The AUC of serum ferritin = 0.540, the AUC of PCT = 0.527, and the AUC of CRP = 0.588, only *p*_*CRP*_ < 0.01.

Procalcitonin and CRP in patients with bacterial co-infection were significantly higher than those in patients with non-bacterial co-infection (both *p* < 0.001, Table 4). The median PCT and CRP levels in 115 patients with bacterial co-infection [2.09 ng/ml (range 0.99–3.6 ng/ml) and 43.2 mg/l (range 20.5–75.3 mg/l)] were higher than those in 258 patients with non-bacterial co-infections [0.99 ng/ml (range 0.63–2.00 ng/ml) and 26.4 mg/l (range 14.2–46.3 mg/l), respectively, both *p* < 0.001] (Table 4). The AUC values of PCT and CRP for predicting bacterial co-infection were 0.527 (95% CI 0.464–0.589, *p* = 0.401) and 0.588 (95% CI 0.525–0.652, *p* = 0.005), respectively (Figure 2 and Table 5). These results showed that CRP can be used as a potential biomarker to predict bacterial co-infection in patients with HFRS.

**TABLE 4:** Demographics and clinical and laboratory data at admission in the patients with and without bacterial infection*.

Variables	Non-bacterial infection (*n* = 258)	Bacterial infection (*n* = 115)	*P*-value
Male, n (%)	199 (77.7)	87 (75.7)	0.7550
Age, years	46 (34–54)	48.1 ± 14.3	0.0143
Time from symptom onset to hospital admission, days	5 (4–7)	5 (4–7)	0.3315
Hospital stays, days	7 (5–9)	9 (6–12)	<0.0001
**Comorbidity**			
Hypertension, n (%)	9 (3.5)	12 (10.4)	0.0072
Coronary heart disease, n (%)	1 (0.4)	3 (2.6)	0.1679
Diabetes, n (%)	5 (1.9)	13 (11.3)	<0.0001
**Complication**			
Hemorrhage, n (%)	18 (7.0)	87 (75.7)	<0.0001
Hepatic injury, n (%)	171 (66.3)	88 (76.5)	0.0752
Myocardial damage, n (%)	104 (40.3)	52 (45.2)	0.4228
CNS damage	0	1 (0.9)	0.3351
ARDS, n (%)	2 (0.78)	17 (14.8)	<0.0001
MODS, n (%)	0	21 (18.3)	<0.0001
Respiratory support, n (%)	2 (0.8)	33 (28.7)	<0.0001
CRRT, n (%)	20 (7.8)	22 (19.1)	0.0013
Number of death, n (%)	0	14 (12.2)	<0.0001
**Parameters**			
WBC, × 10^9^/L	8.9 (5.5–10.8)	8.3 (4.9–11.2)	0.9748
PLT, × 10^9^/L	98.0 (54.0–121.0)	94.5 (50.0–120.0)	0.4762
AST, U/L	98.4 (58.6–183.9)	128.4 (72.1–212.6)	0.0273
ALT, U/L	104.6 (64.0–184.0)	108.0 (74.0–168.0)	0.5610
LDH, U/L	460 (345–657)	489.0 (380.0–765.5)	0.1093
α-HBDH, U/L	333.0 (259.0–496.0)	364.0 (289.0–556.0)	0.0295
PCT, ng/ml	0.99 (0.63–2.00)	2.09 (0.99–3.6)	0.0001
Ferritin, ng/ml	807.85 (515.1–1358.8)	2799.0 (1542.8–5941.7)	0.0001
CRP, mg/l	26.4 (14.2–46.3)	43.2 (20.5–75.3)	0.0001
Lg PCT	−0.004 (−0.2 to 0.3)	0.3 (−0.004 to 0.6)	0.0001
Lg Ferritin	2.9 ± 0.3	3.4 ± 0.4	0.0001
Lg CRP	1.4 ± 0.4	1.6 (1.3–1.9)	0.0001

*ARDS, acute respiratory distress syndrome; MODS, multiple organ dysfunction syndrome; CNS, central nervous system; CRRT, continuous renal replacement therapy; WBC, white blood cell count; PLT, platelet; ALT, alanine transaminase; AST, aspartate aminotransferase; LDH, lactate dehydrogenase; and α-HBDH, α-hydroxybutyrate dehydrogenase; Lg Ferritin, Lg PCT, and Lg CRP, Ferritin, procalcitonin, and CRP after log10 transformation.

**TABLE 5:** Predictive values of parameters for the bacterial infection of HFRS*.

Variables	AUC	*p* value	Cut-off value	Sensitivity	Specificity	95% CI for AUC	
						
						Lower	Upper
Lg PCT	0.527	0.401	0.16	0.66	0.44	0.464	0.589
Lg CRP	0.588	0.005	1.73	0.43	0.76	0.525	0.652
Lg Ferritin	0.540	0.214	3.32	0.758	0.333	0.477	0.602

*AUC, area under the receiver operating characteristic (ROC) curve; CI, confidence interval; Lg PCT, procalcitonin after log10 transformation; Lg CRP, C-reactive protein after log10 transformation; Lg Ferritin, serum ferritin after log10 transformation.

### Serum Ferritin, Procalcitonin, and C-Reactive Protein Association With Patient Survival

Of the 373 patients, 14 patients died of HFRS with a mortality rate of 3.8%. All the non-survivors had a severe hemorrhage, hepatic injury, myocardial damage, and even MODS, and received CRRT treatment and/or respiratory support. The occurrence of ARDS and MODS in non-survivors was significantly higher than those of survivors (both *p* < 0.001, Table 6). Non-survivors had a significantly lower PLT, but higher LDH and α-HBDH (*p* < 0.001, Table 6).

**TABLE 6:** Demographics and clinical and laboratory data at admission in survivors and non-survivors of patients with HFRS*.

Variables	Survivors (*n* = 359)	Non-survivors (*n* = 14)	*P*-value
Male, n (%)	273 (76.0)	13 (92.9)	0.1445
Age, years	46 (35–56)	49 (43–55)	0.5351
Time from symptom onset to hospital admission, days	5 (4–7)	5 (4–7)	0.887
Hospital stays, days	7 (5–10)	13.7 ± 9.0	0.0066
**Comorbidity**			
Hypertension, n (%)	17 (4.7)	4 (28.6)	0.0047
Coronary heart disease, n (%)	1 (0.3)	3 (21.4)	<0.0001
Diabetes, n (%)	16 (4.5)	2 (14.3)	0.1173
**Complication**			
Hemorrhage, n (%)	91 (25.3)	14 (100.0)	<0.0001
Hepatic injury, n (%)	245 (68.2)	14 (100.0)	0.0114
Myocardial damage, n (%)	142 (39.6)	14 (100.0)	<0.0001
ARDS, n (%)	7 (1.9)	12 (85.7)	<0.0001
MODS, n (%)	7 (1.9)	14 (100.0)	<0.0001
Respiratory support, n (%)	27 (0.75)	8 (57.1)	<0.0001
CRRT, n (%)	28 (0.78)	14 (100.0)	<0.0001
**Parameters**			
WBC, × 10^9^/L	8.9 (5.3–11.0)	8.3 (4.9–10.3)	0.6899
PLT, × 10^9^/L	102 (65–121)	29 (10–60)	0.0001
AST, U/L	101.0 (67.9–182.5)	145.2 (72.7–275.0)	0.1812
ALT, U/L	99.7 (60.0–183.9)	172.0 (86.0–306.1)	0.0458
LDH, U/L	443.0 (348.0–623.0)	1343.1 ± 883.4	<0.0001
α-HBDH, U/L	328.0 (263.0–461.0)	698.0 (377.0–1114.0)	0.0001
PCT, ng/ml	1.7 (0.9–3.1)	3.6 (2.2–42.9)	0.0009
Ferritin, ng/ml	3410.0 (1456.9–9028.0)	21708.0 (14101.0–36078.0)	<0.0001
CRP, mg/l	31.2 (17.6–60.1)	65.0 ± 35.5	0.0055
Lg PCT	0.2 (−0.6 to 0.5)	0.8 ± 0.8	0.0009
Lg Ferritin	3.6 ± 0.6	4.3 ± 0.4	<0.0001
Lg CRP	1.5 (1.3–1.8)	1.7 ± 0.3	0.0054

*ARDS, acute respiratory distress syndrome; MODS, multiple organ dysfunction syndrome; CRRT, continuous renal replacement therapy; WBC, white blood cell count; PLT, platelet; ALT, alanine transaminase; AST, aspartate aminotransferase; LDH, lactate dehydrogenase; and α-HBDH, α-hydroxybutyrate dehydrogenase; Lg Ferritin, Lg PCT, and Lg CRP, Ferritin, procalcitonin, and CRP after log10 transformation.

The levels of serum ferritin, PCT, and CRP in non-survivors were significantly higher compared with survivors (all *p* < 0.001, Table 6). The median serum ferritin level of non-survivors [21,708.0 ng/ml (range 14,101.0–36,078.0 ng/ml)] was higher than that in 359 survivors [3,410.0 ng/ml (range 1,456.9–9,028.0 ng/ml)] (*p* < 0.001, Table 6). The median PCT [3.6 ng/ml (range 2.2–42.9 ng/ml)] and CRP [50.4 mg/l (range 29.5–100.5 mg/l)] levels of non-survivors was higher than those in 359 survivors [1.7 ng/ml (range 0.9–3.1 ng/ml) and 31.2 mg/l (range 17.6–60.1 mg/l)], respectively (*p* < 0.001, Table 6).

**FIGURE 3:**
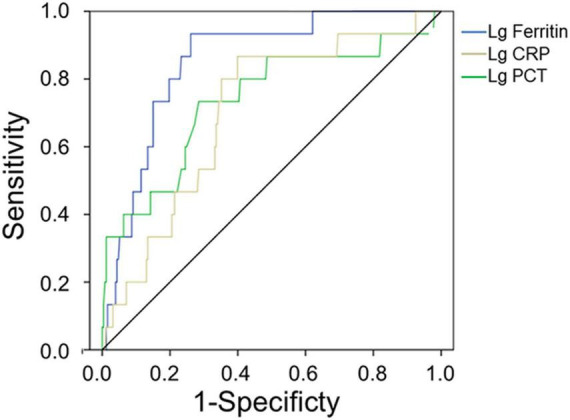
Receiver operating characteristics curve showing the performance of serum ferritin, PCT, and CRP as predictors of mortality. The AUC of serum ferritin = 0.853, the AUC of PCT = 0.737, and the AUC of CRP = 0.703, only *p*_*Serum ferritin*_ < 0.001.

The AUC values of serum ferritin, PCT, and CRP for predicting mortality were 0.853 (95% CI 0.774–0.933, *p* < 0.001), 0.737 (95% CI 0.587–0.887, *p* = 0.002), and 0.703 (95% CI 0.577–0.828, *p* = 0.008), respectively (Figure 3 and Table 7), showing that serum ferritin was the best marker to predict mortality of HFRS.

**TABLE 7:** Predictive values of parameters for the mortality of HFRS*.

Variables	AUC	*p* value	Cut-off value	Sensitivity	Specificity	95% CI for AUC	
						
						Lower	Upper
Lg PCT	0.737	0.002	0.50	0.733	0.715	0.587	0.887
Lg CRP	0.703	0.008	1.62	0.867	0.601	0.577	0.828
Lg Ferritin	0.853	<0.001	4.03	0.933	0.739	0.774	0.933

*AUC, area under the receiver operating characteristic (ROC) curve; CI, confidence interval; Lg PCT, procalcitonin after log10 transformation; Lg CRP, C-reactive protein after log10 transformation; Lg Ferritin, serum ferritin after log10 transformation.

## Discussion

This study analyzed the changes of serum ferritin, PCT, and CRP in patients with HFRS and compared their clinical significance for the prediction of severity and mortality in patients with HFRS. Results showed that serum ferritin and PCT concentrations have a robust association with severity and mortality of HFRS, which can be used as promising predictors of severity and mortality, and CRP may be an effective biomarker to assess bacterial co-infection in HFRS.

High levels of serum ferritin have been detected in patients of acute viral infections, such as Epstein-Barr virus, human immunodeficiency virus, and dengue virus, and it is associated with the severity and poor prognosis of virus infection (Riera et al., 1994; Mustafa et al., 2001; van de Veerdonk et al., 2012; Soundravally et al., 2015). Serum ferritin is associated with viremia and is significantly elevated in patients with hemorrhagic manifestation and the fatal outcome of the Ebola virus (McElroy et al., 2014; van der Ven et al., 2015). Recent studies have shown that serum ferritin is also a prognostic biomarker that contributes to therapeutic decision-making concerning patients with COVID-19 (Cooper et al., 2020; Kappert et al., 2020). Our results have demonstrated that serum ferritin is a potential biomarker to predict the severity of patients with HFRS, with the cutoff value of 3,548 ng/ml in serum concentration.

Procalcitonin and CRP are considered the most sensitive and effective biomarkers to assess the severity of bacterial co-infection or sepsis (Hu et al., 2017; Cui et al., 2019). Recently, PCT has been reported to be associated with the severity and prognosis of the hantavirus infection (Fan X. et al., 2018) and COVID-19 (Guan et al., 2020; Huang et al., 2020; Lu et al., 2020; Zhang et al., 2020). In the present study, we compared the clinical significance of serum ferritin, PCT, and CRP in patients with HFRS, and found that they were all associated with mortality, but the serum ferritin had better performance than both PCT and CRP to predict the prognosis of HFRS.

Procalcitonin is also shown to be associated with the severity and prognosis of HFRS, as PCT greater than 2.8 ng/ml suggested a severe virus infection and more than 3.16 ng/ml indicated a very poor prognosis. In patients with HFRS with bacterial co-infection, PCT began to rise in the viremia stage, so PCT is unable to distinguish between viral and bacterial co-infections. In contrast, the level of CRP was normally or slightly elevated in hantavirus infection, but it was significantly increased in patients with HFRS with bacterial co-infection, which was related to the severity of infection and prognosis. Therefore, it can be used in the evaluation of bacterial co-infection and prognosis in patients with HFRS.

## Conclusion

The study showed that serum ferritin and PCT have a robust association with the severity and mortality of HFRS, which can be used as a promising predictor of severity and mortality in rodent-borne disease. CRP may be an effective biomarker to assess bacterial co-infection in HFRS.

## Data Availability Statement

The original contributions presented in the study are included in the article/supplementary material, further inquiries can be directed to the corresponding author/s.

## Ethics Statement

The studies involving human participants were reviewed and approved by the Ethics Committee of The First Hospital of Jilin University. The patients/participants provided their written informed consent to participate in this study.

## Author Contributions

LC and QL conceived the project. LC planned the study. ND, LL, YZ, and KZ collected the data. LC, ZW, and QL analyzed the data and drafted the manuscript. All authors were involved in critically revising the manuscript and approving the final version.

## Conflict of Interest

The authors declare that the research was conducted in the absence of any commercial or financial relationships that could be construed as a potential conflict of interest.

## Publisher’s Note

All claims expressed in this article are solely those of the authors and do not necessarily represent those of their affiliated organizations, or those of the publisher, the editors and the reviewers. Any product that may be evaluated in this article, or claim that may be made by its manufacturer, is not guaranteed or endorsed by the publisher.
